# Life-Threatening Autoimmune Hemolytic Anemia and Idhiopatic Thrombocytopenic Purpura. Successful Selective Splenic Artery Embolization

**DOI:** 10.4084/MJHID.2016.020

**Published:** 2016-04-10

**Authors:** Matteo Molica, Fulvio Massaro, Giorgia Annechini, Erminia Baldacci, Gianna Maria D’Elia, Riccardo Rosati, Silvia Maria Trisolini, Paola Volpicelli, Robin Foà, Saveria Capria

**Affiliations:** 1 Department of Cellular Biotechnologies and Hematology, Sapienza University, Rome, Italy; 2 Department of Radiological Sciences, Vascular and Interventional Unit, “Sapienza” University of Rome, Rome, Italy

## Abstract

Selective splenic artery embolization (SSAE) is a nonsurgical intervention characterized by the transcatheter occlusion of the splenic artery and/or its branch vessels using metallic coils or other embolic devices. It has been applied for the management of splenic trauma, hypersplenism with portal hypertension, hereditary spherocytosis, thalassemia and splenic hemangioma. We hereby describe a case of a patient affected by idiopathic thrombocytopenic purpura (ITP) and warm auto-immune hemolytic anemia (AIHA) both resistant to immunosuppressive and biological therapies, not eligible for a surgical intervention because of her critical conditions. She underwent SSAE and achieved a hematologic complete response within a few days without complications. SSAE is a minimally invasive procedure to date not considered a standard option in the management of AIHA and ITP. However, following the progressive improvement of the techniques, its indications have been extended, with a reduction in morbidity and mortality compared to splenectomy in patients with critical clinical conditions. SSAE was a lifesaving therapeutic approach for our patient and it may represent a real alternative for the treatment of resistant AIHA and ITP patients not eligible for splenectomy.

## Introduction

Auto-immune hemolytic anemia (AIHA) is a clinical condition in which IgG autoantibodies bind to red blood cell (RBC) surface antigens, initiating destruction via the complement and reticuloendothelial system. The disorder can be idiopathic, but in approximately 50% of cases it is associated with an underlying condition, as lymphoproliferative and connective tissue diseases. In particular, treatment of warm AIHA relies on transfusions, corticosteroids, other immunosuppressive agents such as azathioprine and cyclophosphamide or more recently on rituximab. Splenectomy is usually considered the second line treatment in surgical candidates with life threatening AIHA in whom immunosuppressive therapy is unsuccessful. The surgical approach has many disadvantages, such as high invasiveness, increased blood loss and severe operative and post-operative infective complications,^1^ thus it is contraindicated in selected high risk patients. With the development of interventional radiology, the use of selective splenic artery embolization (SSAE) in the clinical practice has greatly extended. SSAE is a minimally invasive procedure consisting in the trans catheter occlusion of the splenic artery and/or its branch vessels using metallic coils or other embolic devices and associated with few side effects: the most significant is severe pain radiating from the left upper quadrant, presumably from nerve endings in the splenic capsule, and it requires medication, including opiates, which, if given in time, can prevent splinting. Common indications of SSAE include hypersplenism with portal hypertension, hereditary spherocytosis, thalassemia, splenic trauma, splenic hemangioma, and liver cancer.^2^ The SSAE is not indicated as upfront therapy in patients with either warm or cold AIHA. However, it might be considered for the treatment of selected patients with life-threatening AIHA and idiopathic thrombocytopenic purpura (ITP) not eligible for splenectomy and highly resistant to conventional therapies.^3^,^4^ The same applies for paroxysmal nocturnal hemoglobinuria (PNH) according to the results of a recently published paper.^5^ We describe a case of a young woman affected by resistant AIHA and ITP who, in view of her critical conditions related to anemia and piastrinopenia, was submitted to SSAE which was followed by a rapid and significant hematological improvement of both diseases.

## Case Report

**Figure 1:**
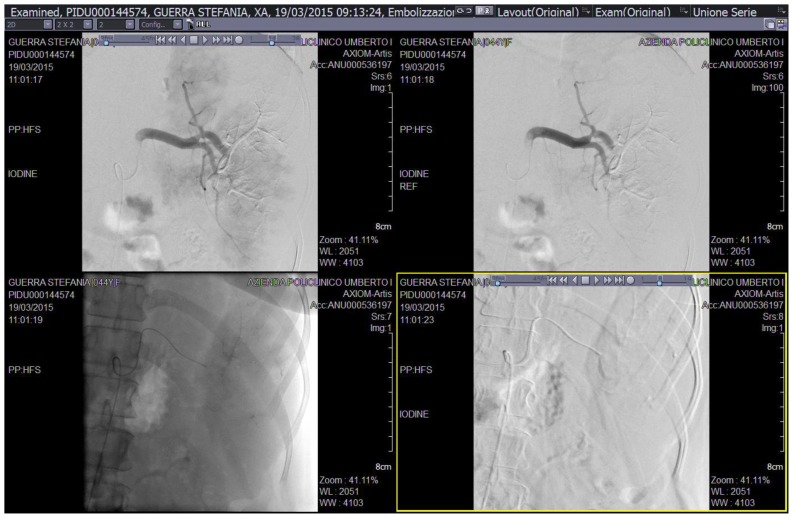
SSAE of the inferior branches of the splenic artery.

**Figure 2:**
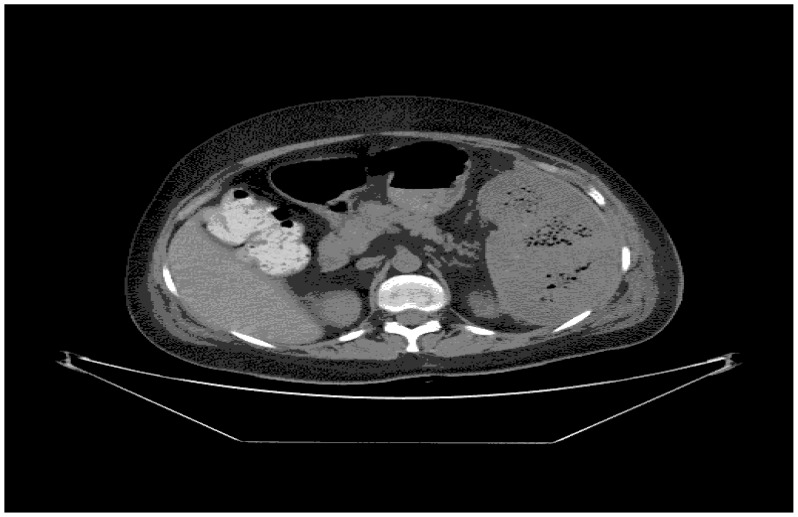
CT scan showing multiple infarcts in the parenchyma of the spleen.

**Figure 3:**
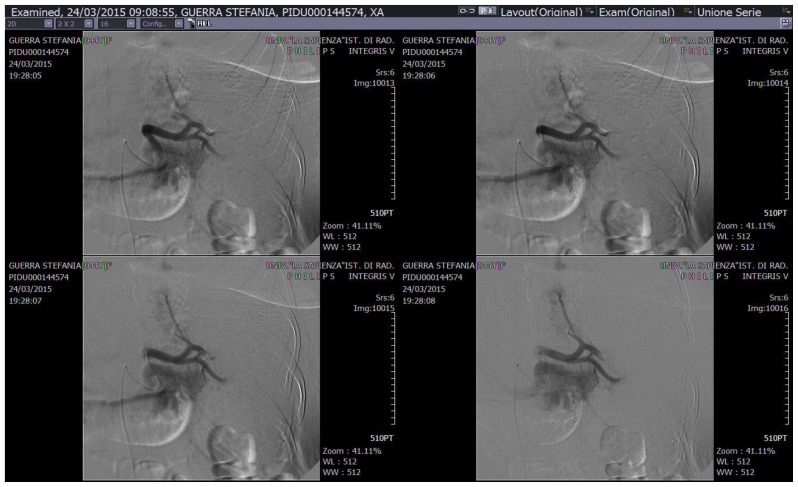
SSAE of the the two remaining major branches of the splenic artery.

**Figure 4:**
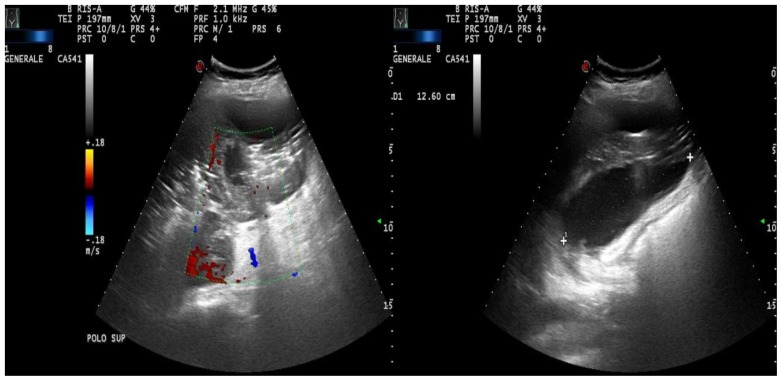
Abdomen ultrasound showing a decrease of the spleen volume and avascularization of the spleen after SSAE.

**Figure 5:**
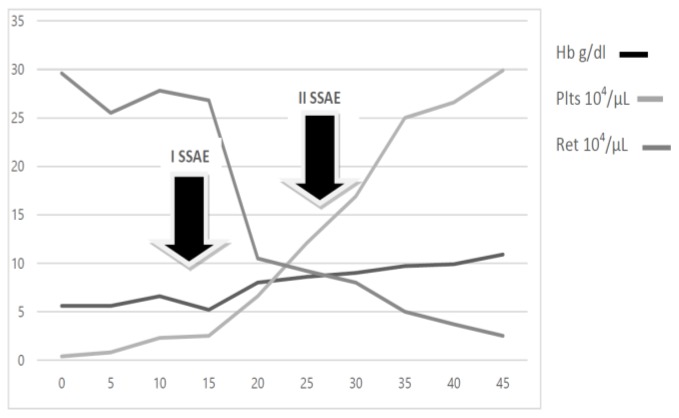
Time course of hematological parameters of patient in relation to SSAE treatment.

**Figure 6:**
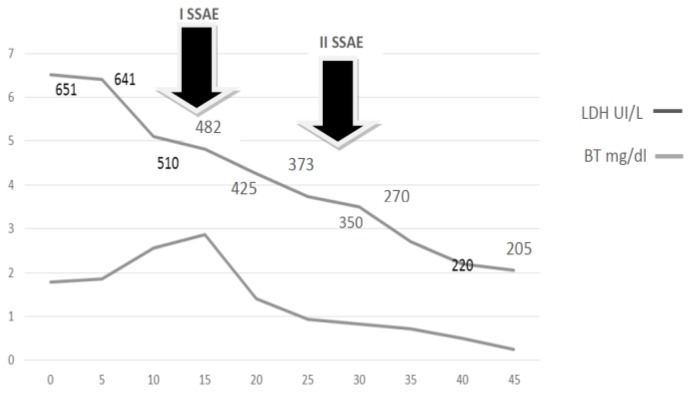
Time course of hemolysis indices of patient in relation to SSAE treatment

A 43-year old woman was diagnosed with stage IIE bulky Hodgkin’s lymphoma at our Hematologic Center in May 2013. She was treated with four cycles of chemotherapy with doxorubicin, bleomycin, vinblastine, dacarbazine (ABVD), followed by 30 Gy radiotherapy to the mediastinum, left laterocervical and sovraclaveaer nodes districts. The patient achieved a complete remission after two cycles of ABVD and remains in ongoing complete remission as evidenced by CT/PET. About six months after the end of therapy, the patient suddenly developed a severe thrombocytopenia (7×10^3^/μL) burdened by persistent metrorrhagia and a progressive anemia, initially interpreted as secondary to bleeding. The patient was immediately subjected to a total body CT scan, a bone marrow aspirate with cytogenetic analysis and a bone marrow biopsy that definitely ruled out a relapse of Hodgkin’s lymphoma or an early development of a myelodisplastic syndrome chemotherapy/radiotherapy-related. Despite the intravenous administration of human immunoglobulins at the standard dose of 1 g/Kg and high dose methilprednisolone (80 mg/day), thrombocytopenia persisted. Treatment with eltrmbopag was given for two weeks at a standard dose of 50 mg/day, but it was suddenly stopped because of grade 3 WHO liver toxicity. Moreover, a further worsening of anemia (Hb 8 g/dl) was observed, with a progressive increase of hemolysis indices: LDH 425 UI/L (UNL 225 UI/L), total bilirubin 2.3 mg/dl (normal range 0.3–1.0 mg/dl). A peripheral blood film was consistent with warm AIHA (i.e., presence of an increased number of spherocytes and polychromatic red cells) and a direct antiglobulin test showed strong agglutination to anti-IgG and weak reactivity to anti-C3. The eluate was equally positive against all cells tested. As salvage therapy, the patient received two weekly administrations of Rituximab at the standard dose of 375 mg/m^2^, stopped due to cytomegalovirus infection reactivation that further worsened the peripheral blood pancytopenia and the patient’s clinical conditions. The patient also began immunosuppressive treatment with azathioprine at the dose of 100 mg/day, but despite a constant transfusion support, the general conditions rapidly worsened, the patient became symptomatic secondary to tissue hypoxia. Physical examination revealed tachycardia (130 beats/min), tachypnea (22 breaths/min), mild hypotension (90/50 mm Hg) and a markedly depressed level of consciousness. The CBC showed hemoglobin 4.8 g/dL, platelets 5×10^3^/μL and blood chemistry revealed a LDH level of 687 UI/l. Because splenectomy would have exposed the patient to a high surgical risk we considered SSAE as a possible therapeutic approach. Therefore, the patient underwent selective embolization of the inferior branches of the splenic artery, using gelfoam pledgets and microcoils (Figure 1). Except for a diffuse abdominal pain which subsided within 48 hours, the patient tolerated the procedure well. The abdominal CT scan performed two days after SSAE showed an important decrease of the spleen volume with multiple infarcts in the mid to lower pole of the spleen (Figure 2). Hemoglobin rose to 9.7 g/dl with a gradual decrease of hemolysis indices (LDH 370 UI/L, total bilirubin 1.4 mg/dl) and the platelet count significantly recovered, stabilizing at 60–80 × 10^3^/μL. Ten days later, a second SSAE procedure was carried out: this time the two remaining major branches of the splenic artery were embolized (Figure 3), leading to a complete exclusion of the organ from the bloodstream. Hemoglobin increased to 10.9 g/dl with a resolution of hemolysis (LDH 205 UI/L; total bilirubin 0.50 mg/dl; normal reticulocyte count) and the platelet count reached 296 × 10^3^/μL. An abdominal ultrasound exam performed two days after the embolization, demonstrated a marked splenic decrease and a complete avascularization of the organ (Figure 4). After two months of hospitalization, the patient was finally discharged. After four months from SSAE, she retains a good control of the blood counts with no signs of hemolysis (Figures 5 and 6).

## Discussion

According to current guidelines,^6^ our patient - resistant to intravenous immunoglobulins, steroids, rituximab and azathioprine therapy - would have undergone splenectomy, the only therapeutic approach potentially capable of blocking the uncontrollable hemolysis. Removal of the spleen has a two-fold effect: it removes the primary site of extravascular hemolysis and a site of antibody production. The patient’s poor clinical conditions and the extremely low platelet values that would have exposed the patient to a high intrasurgery risk of bleeding, prompted us to attempt - as salvage treatment - a SSAE performed in two times to conclusively exclude the organ from the bloodstream avoiding spleen mediated hemolysis. Such a therapeutic approach is available since the 1970’s, when Spigos et al used it to treat renal transplant patients with hypersplenism and persistent leukopenia.^7^ In resistant AHIA and ITP, this procedure may have an important role when a splenectomy is impossible to carry out due to the high surgical risks related to the low hemoglobin and/or platelet values. Furthermore, SSAE may represent a first step allowing a complete recovery of blood parameters after which it may become possible to perform a splenectomy with less intra- and post-surgery risks. The patient underwent a partial, followed by near-total, splenic artery embolization and experienced a sustained remission of AIHA and a complete platelet recovery, reflecting the block of the hemolysis. Splenic embolization proved to be a lifesaving treatment and represented a safe and effective therapeutic approach in this extreme emergency.

## References

[1] Leone G, Pizzigallo E (2015). Bacterial infections following splenectomy for malignant and nonmalignant hematologic diseases. Mediterr J Hematol Infect Dis.

[2] Guan YS, Hu Y (2014). Clinical application of partial splenic embolitazion. The Scientific World Journal.

[3] Campbell R, Marik PF (2005). Severe autoimmune hemolytic anemia treated by paralysis, induced hypotermia and splenic embolization. Chest.

[4] Miyazaki M, Itoh H, Kaiho T, Ohtawa S, Ambiru S, Hayashi S, Nakajima N, Oh H, Asai T, Isek T (1994). Partial splenic embolization for the treatment of chronic idiopathic thrombocytopenic purpura. American Journal of Roentgenology.

[5] Araten DJ, Iori AP, Brown k, Torelli GF, Barberi W, Natalino F, De Propris MS, Girmenia C, Salvatori FM, Zelig O, Foà R, Luzzatto L (2014). Selective splenic artery embolization for the treatment of thrombocytopenia and hypersplenism in paroxysmal nocturnal hemoglobinuria. J Hematol Oncol.

[6] Barcellini W, Fattizzo B, Zaninoni A, Radice T, Nichele I, Di Bona E, Lunghi M, Tassinari C, Alfinito F, Ferrari A, Leporace AP, Niscola P, Carpenedo M, Boschetti C, Revelli N, Villa MA, Consonni D, Scaramucci L, De Fabritiis P, Tagariello G, Gaidano G, Rodeghiero F, Cortelezzi A, Zanella A (2014). Clinical heterogeneity and predictors of outcome in primary autoimmune hemolytic anemia: a GIMEMA study of 308 patients. Blood.

[7] Spigos D, Jonasson O, Mozes M, Capek V (1979). Partial splenic embolization in the treatment of hypersplenism. American Journal of Roentgenology.

